# Testing for coeliac disease rarely leads to a diagnosis: a population-based study

**DOI:** 10.1080/02813432.2021.1935045

**Published:** 2021-06-17

**Authors:** Maxine D. Rouvroye, Lotte Oldenburg, Pauline Slottje, Johanna H. K. Joosten, Renee X. de Menezes, Marcel E. Reinders, Gerd Bouma

**Affiliations:** a Department of Gastroenterology and Hepatology, Amsterdam Gastroenterology Endocrinology Metabolism, Amsterdam UMC, Vrije Universiteit Amsterdam, Amsterdam, Netherlands; b Department of General Practice, Amsterdam Public Health Research Institute, Amsterdam UMC, Amsterdam, The Netherlands; c Department of General Practice, Academic Network of General Practice, Amsterdam UMC, Amsterdam, The Netherlands; d Department of Epidemiology and Biostatistics, Amsterdam UMC, Vrije Universiteit Amsterdam, Amsterdam, The Netherlands

**Keywords:** Coeliac disease, diagnosis, gastroenterology, general practice, irritable bowel syndrome, transglutaminase 2

## Abstract

**Background:**

Coeliac disease (CD) has an estimated prevalence of ∼1% in Europe with a significant gap between undiagnosed and diagnosed CD. Active case finding may help to bridge this gap yet the diagnostic yield of such active case finding in general practice by serological testing is unknown.

**Objective:**

The aim of this study was to determine (1) the frequency of diagnosed CD in the general population, and (2) to investigate the yield of active case finding by general practitioners.

**Methods:**

Electronic medical records of 207.200 patients registered in 49 general practices in The Netherlands in 2016 were analysed. An extensive search strategy, based on International Classification of Primary Care codes, free text and diagnostic test codes was performed to search CD- or gluten-related contacts.

**Results:**

The incidence of CD diagnosis in general practice in 2016 was 0.01%. The prevalence of diagnosed CD reported in the general practice in the Netherlands was 0.19%, and considerably higher than previously reported in the general population. During the one year course of the study 0.95% of the population had a gluten-related contact with their GP; most of them (72%) were prompted by gastrointestinal complaints. Serological testing was performed in 66% (*n* = 1296) of these patients and positive in only 1.6% (*n* = 21).

**Conclusion:**

The number of diagnosed CD patients in the Netherlands is substantially higher than previously reported. This suggests that the gap between diagnosed and undiagnosed patients is lower than generally assumed. This may explain that despite a high frequency of gluten-related consultations in general practice the diagnostic yield of case finding by serological testing is low.

**Key points**
- The diagnostic approach of GPs regarding CD and the diagnostic yield is largely unknown
- Case finding in a primary health care practice has a low yield of 1.6%
- CD testing was mostly prompted by consultation for gastrointestinal symptoms
- There is a heterogeneity in types of serological test performed in primary care

## Background

Coeliac disease (CD) is a chronic, immune-mediated enteropathy in individuals with a genetic predisposition, which is triggered by the ingestion of gluten. Its appraised prevalence in the general population in Europe is 1% but varies considerably between populations [1–3]. Historically, CD was thought of as a paediatric disorder, but in the last decades this dogma was revaluated with a significant proportion of patients diagnosed above the age of 40 [4].

It has been estimated that for each diagnosed CD case there are five to 10 undiagnosed CD patients, also referred to as the CD iceberg, generating the impression of a gap between diagnosed and undiagnosed CD [5–8]. General practitioners (GPs) act as gatekeepers and are the first in line of the diagnostic process of CD. Almost all non-institutionalized citizens are enlisted at a general practice in the Netherlands. Current guidelines (mainly developed by and for medical specialists) advocate a policy of active case-finding in CD [9]. How these guidelines are implemented in daily care by GPs is unknown. The general impression among GPs is that regardless of frequent serological testing, CD is rarely diagnosed [10].

The aim of the present study was twofold. First, we wanted to better define the gap between diagnosed and undiagnosed CD patients. Hereto, we determined the number of established diagnoses in an extensive cohort of patients representing the general population and compared these data with established data on the seroprevalence of CD. Second, we wanted to study which symptoms trigger GPs to test for CD and what diagnostic approach they follow. Such information may help to breach the gap between diagnosed and undiagnosed CD.

## Methods

### Patient selection and data extraction

For this observational study, we used anonymized patient data extracted from the database of the Academic Network of General practice at Amsterdam UMC, location VU medical centre (ANH VUmc). Our target population consisted of patients with a gluten- or CD-associated contact and/or gluten- or CD-related lab result in 2016 plus those who were registered to have CD previously diagnosed based on ICPC-1 D99.06. The ANH VUmc database encompasses pseudonimised electronic medical record data from over 49 general practices in and near the city of Amsterdam.

**Figure 1.**
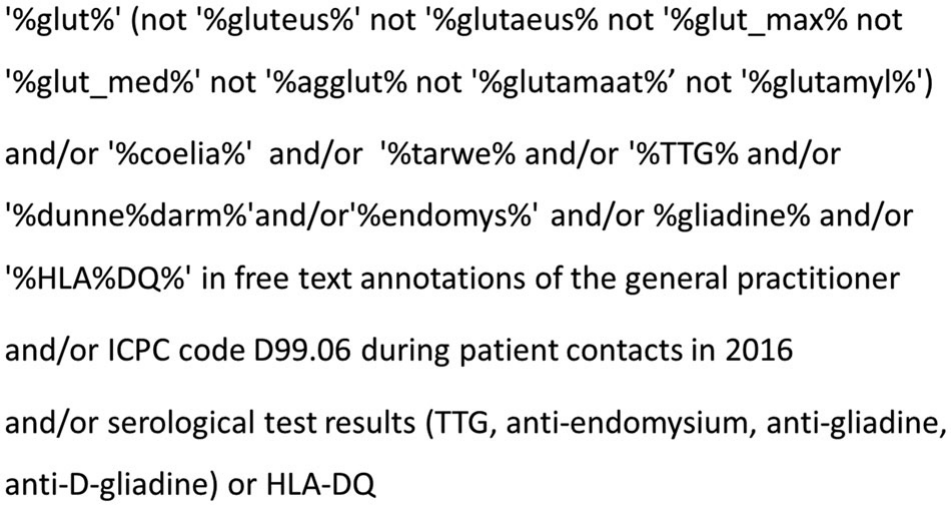
Figure one depicts the search string that was used to find eligible cases. Three techniques were used that to find cases both separate from each other or combined (and/or). In free text annotations, in the ICPC coding system and in laboratory test results. TTG: tissue Transglutaminase, HLA: human leukocyte antigen, ICPC: International Classification of Primary Care, D99.06: code for coeliac disease diagnosis.

An extensive three-track search strategy was built to maximize the chance of finding our target population. All records of patients registered in these general practices in the year 2016 were scrutinized for the International Classification of Primary Care (ICPC-1) code Coeliac Disease (D99.06); the terms ‘coeliac disease’, ‘gluten’ in free text annotations of the general practitioner during patient contacts in the year 2016; and serological test results (TTG, anti-endomysium, anti-gliadine, anti-D-gliadine) or HLA-DQ in the year 2016. See Figure 1 for search string details. All patients that came up in at least one of those three search options were further investigated.

All general practice contacts (i.e. consultations, telephone calls, home visits and other contacts such as e-consultations), and all laboratory results of the selected patients were reviewed by one author (LO) to decide upon eligibility for the analytical sample (i.e. patient with a gluten- or CD-associated contact and/or laboratory test results in 2016, plus those who were registered to have CD previously based on ICPC-1 D99.06 [11]). A second reviewer (MDR) scrutinized 40% of the data to assess inter-investigator variation. Initial inconsistencies in eligibility decisions between these two reviewers were resolved by a third author (MER). Of this target population the following data were collected from coded and free text annotations in the CD/gluten-related consultations in 2016, laboratory results and medical history: clinical presentation, diagnosis and serological testing and results, dietary behaviour, family history on CD and referrals in cases with laboratory test results in January 2016, the data from the initial consultation in 2015 was also reviewed. Vice versa, test results after 2016 were requested for patients that were tested for CD in December 2016.

To estimate the frequency or prevalence of diagnosed CD the electronic records and problem lists of all patients that were registered in these general practices in 2016 were screened for ICPC-1 D99.06, regardless of the date of diagnosis, or whether they had a CD-related consult in 2016. Several variables were assessed to see if they could be a possible prognostic risk factor for the diagnosis of CD (e.g. type of chief complaint, relatives with CD, active test request by patient, gender, age, thyroid disease).

#### Data analysis

Descriptive analysis was performed using SPSS 22.0. To compare two groups, the Mann-Whitney-u test was used for continuous variables, whereas the chi-square test or a Fisher’s exact test for smaller subgroup sizes, were used for categorical variables. Observations with missing data were excluded. In all statistical tests a p-value of 0.05 or lower was considered significant. The nominator of the incidence of registered CD in general practice was calculated based on the number of patients that were diagnosed in 2016, i.e. the starting date of ICPC1-code D99.06 was in 2016. The nominator of the prevalence of CD was calculated based on the number of all patients registered in 2016 in these general practices having CD (ICPC-1 D99.06). The denominator in both the incidence and the prevalence was the total number of patients that were registered in the year 2016 in these general practices.

### Ethical approval

This study was approved by the steering committee of the Academic Network of General Practice VUmc, Amsterdam. The ANH database is run according to Dutch privacy legislation and contains pseudonymised general practice care data from all patients of the participating general practices, excluding those patients who object to this. Observational studies based on anonymized data from the ANH VUmc database are exempted from informed consent of patients and do not fall under the remit of the Medical Research Involving Human Subjects Act (in Dutch: Wet medisch-wetenschappelijk onderzoek met mensen (WMO)).

## Results

### Frequency of diagnosed coeliac disease in the general population

Records from a total of 207.200 patients were analysed. Among these, 402 patients were registered with the ICPC-1 code for coeliac disease (D99.06), corresponding to a prevalence of 194 registered CD cases per 100.000 registered patients in the general population (0.19%) in 2016.

### Incidence of reported coeliac disease

**Figure 2.**
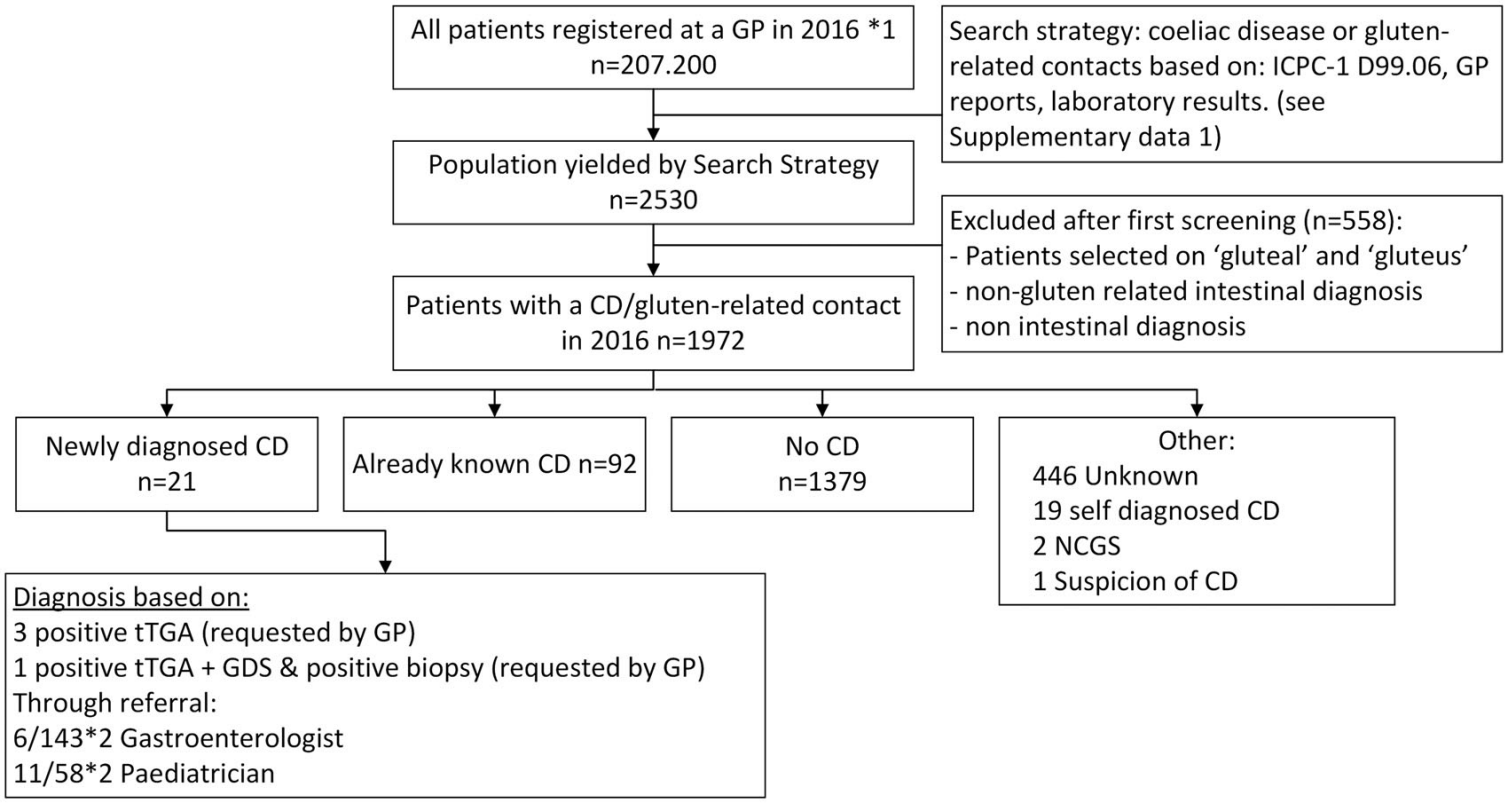
Flowchart of search strategy and patient selection based on general practice contacts in 2016 related to gluten or coeliac disease in a cohort of 207.200 patients distributed over 49 general practices and distribution of diagnosis in the included study population (*n* = 1972). ICPC-1: International Classification of Primary Care, D99.06: ICPC-1 code for coeliac disease, GP: general practitioner, CD: coeliac disease, NCGS: non-coeliac gluten sensitivity, tTGA: tissue Transglutaminase antibodies, GDS: gastroduodenoscopy. *1: Number of patients registered in 49 of the general practices associated with the Academic network of general practice at VU University Medical Centre in 2016, *2 patients with a confirmed diagnosis out of all patients that were referred to a specialist.

As depicted in Figure 2; 21 patients were newly diagnosed with CD in general practice during the one year study period. Eleven of the newly diagnosed patients were referred to a paediatrician, 6 to a gastroenterologist. The incidence of CD was calculated as 21/207.200 per 100.000 patients registered in general practice per year which translates to an incidence of diagnosed CD of 0.01%. Thirteen of these newly diagnosed CD patients were <18 years of age (*p* < 0.001 as compared with adults) and 3 reported having a family member with CD.

### Diagnostic approach of general practitioners

General practitioners are crucial in identifying patients with (potential) CD. Therefore, we next determined the diagnostic attitude of GPs towards (diagnosing) CD. Hereto, an extensive search was done in all patient contacts in the year 2016 in the target population. Figure 2 shows a flowchart of the study population selection. The primary search yielded 2530 patients that potentially had a contact moment with their GP related to gluten or CD. All of the potentially relevant contacts were further analysed; 558 cases were excluded because of incorrect inclusion based on search terms (e.g. gluteal instead of gluten), other intestinal problems or problems not related to CD or gluten.

Baseline characteristics of the remaining 1972 patients (0.95% of the study population) are listed in Table 1. The majority of patients was female (67%) and the median age was 29 years-old (interquartile range (IQR) 20-41).

**Table 1.** Baseline characteristics of all patients (*n* = 1972) with at least one coeliac disease or gluten-related general practice contact in 2016.

Variables	Study population (gluten- or coeliac disease related contact in 2016 (n)	%	Serological test performed in observation period (n)	%	Newly diagnosed coeliac disease (n)	%
Patients	1972	100	1305 ^a^	65.9	21	1.1
Gender, female	1326	67.2	867	66.4	13	61.9
Age, mean (95% CI)^b^	30.8 (13.3–48.0)		31.5 (30.6–32.4)		21.3 (12.3–30.3)	
<22	536	27.2	328	25.1	13	61.9
22-30	545	27.6	359	27.5	1	4.8
31-45	525	26.6	368	28.2	4	19.0
>46	366	18.6	250	19.2	3	14.3
Reported relatives with coeliac disease	65	3.3	46	3.5	3	14.3

In the second column all patients in that group that were serologically tested for coeliac disease (*n* = 1305) are characterised. The last column exhibits the characteristics of all patients that tested positive for CD (*n* = 21).

^a^Including five patients that were previously diagnosed with coeliac disease; CI: confidence interval, ^b^age at time of contact based on year of birth, CD: coeliac disease.

The main symptoms or signs that prompted patients to visit the GP are summarized in Table 2.

**Table 2.** Recorded main reason for CD-or gluten related contact in 2016, and the frequency of serological testing and positive test results in the study population (*n* = 1972).

Reported main reason for consultation						
	Total = 1972		serologically tested = 1305		Positive test result = 21	
	*N*	%^a^	*N*	%^a^	*N*	%
Gastrointestinal	1418	71.9	964	74.8	14	4.3
Stomach ache/cramps	600	30.4	409	68.2	8	2
Diarrhoea	264	13.4	178	67.4	1	0.6
Changing bowel habits	145	7.4	104	71.7	2	1.9
Distension	143	7.3	98	68.5		0
Constipation	81	4.1	52	64.2		0
Non-specific	68	3.4	48	70.6	1	2.1
Nausea	43	2.2	31	72.1	1	3.2
Flatulence	40	2	26	65		0
Rectal blood loss	23	1.2	9	39.1		0
Reflux	5	0.3	3	60		0
Vomiting	2	0.1	2	100	1	50
Borborygmi	2	0.1	2	100		0
Fecal incontinence	1	0.1	1	100		0
Viscous stool	1	0.1	1	100		0
General symptoms	201	10.2	132	50.1	2	0.5
Fatigue	114	5.8	77	67.5		0
Weight loss	30	1.5	23	76.7	2	8.7
Anaemia	26	1.3	15	57.7		0
Weight gain	8	0.4	6	75		0
Growth retardation	8	0.4	2	25		0
Dizziness	2	0.1	2	100		0
Syncope	2	0.1	1	50		0
General malaise	2	0.1	2	100		0
Arthralgia	2	0.1	2	100		0
Excessive Perspiration	1	0.1	1	100		0
Insomnia	1	0.1	0	0		0
Vitamin B deficiency	1	0.1	0	0		0
Hematuria	1	0.1	1	100		0
Angioedema	1	0.1	0	0		0
Asthmatic complaints	1	0.1	0	0		0
Dental problems	1	0.1	0	0		0
Neurological	12	0.6	9	46.3		0
Headache	9	0.5	8	88.9		0
Tingling sensations	2	0.1	1	50		0
Neuropathy	1	0.1	0	0		0
Dermatological	63	3.2	34	60.2		0
Pruritus	23	1.2	14	60.9		0
Eczema	22	1.1	9	40.9		0
Aphthous stomatitis	5	0.3	3	60		0
Other oral cavity abnormalities	4	0.2	2	50		0
Acne	2	0.1	0	0		0
Psoriasis	2	0.1	1	50		0
Exanthema	2	0.1	1	50		0
Rosacea	1	0.1	1	100		0
Alopecia	1	0.1	1	100		0
Lichen Sclerosus	1	0.1	1	100		0
Exanthema	2	0.1	1	50		0
Reported Family History CD	40	2	29	72.5	2	6.9
Fear of CD	20	1	14	70		
Hypothyroid Disease	10	0.5	8	80		
Ophthalmic	3	0.2	3	100		
Not reported	205	10.4	113	55.4	3	2.8
Total	1972	100	1305	66.3	21	1.6

^a^Percentage of total number of patients with CD or glutenrelated contacts for this registered main reason for contact in 2016; CD: coeliac disease.

A total of 1587 serological tests were performed in 1305 patients and included 1282 transglutaminase 2 (tTGA) antibodies, 76 Endomysium antibodies, 12 gliadin antibodies, 217 deamidated gliadin antibodies.

**Figure 3.**
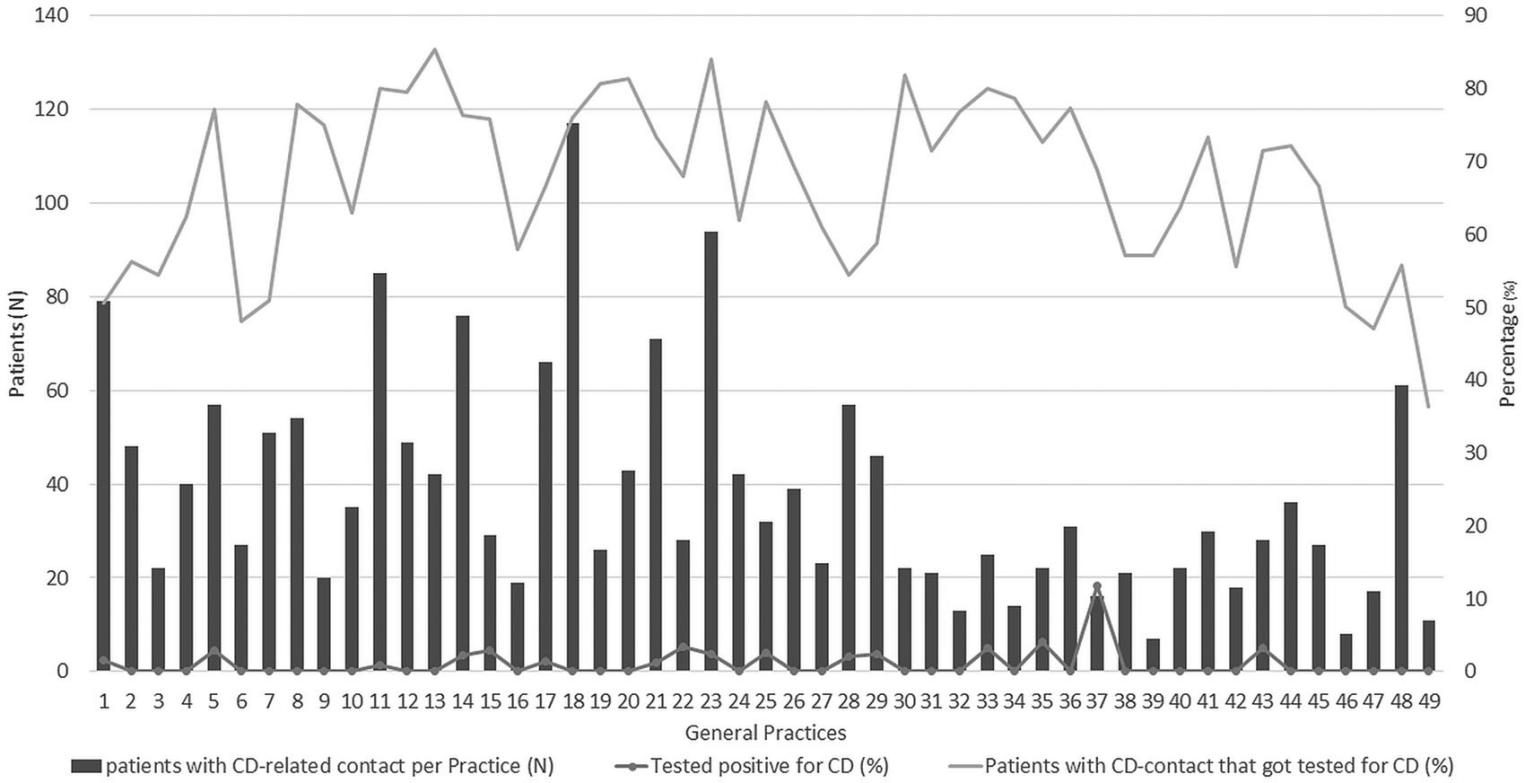
Heterogeneity between general practices (*n* = 49) in the numbers of patients with at least one coeliac disease or gluten-related contact in 2016 and in the diagnostic approach. Patients that were diagnosed before 2016 were excluded from analysis. On the horizontal axis the participating practices are numbered arbitrarily from 1 to 49. The left vertical axis shows the number of patients that had at least one CD-related contact in 2016. The right vertical axis depicts the percentage of these patients that got tested for CD (orange/upper line) in 2016, and the percentage of these tested patients that were diagnosed with CD (black/lower dotted line). CD: coeliac disease.

There was a considerable heterogeneity in the frequency of testing and performed tests between general practices (Figure 3). Whilst tTGA antibodies are most sensitive and specific for screening, a substantial number of deamidated gliadin antibodies (*n* = 217), or gliadin antibodies (*n* = 12) tests were performed. Based on free text annotations, 371 patients actively requested a CD serology test, of whom 254 (68.5%) were tested.

Thyroid disease is known to be more prevalent in individuals with CD [12]. Sixty-three of the included 1972 (3.2%) patients were reportedly known with thyroid disease, which is similar to the 3.05% found in the general population in Europe [13]. 56 (88.9%) thyroid disease patients were tested for CD; they all tested negative.

Based on the free text annotations in the medical records, 242/1972 (12.3%) patients were referred to another specialist; 157/242 (64.9%) to a gastroenterologist, 60 (24.8%) to a paediatrician, 9 (3.7%) to an orthomolecular therapist, 8 (3.3%) to an internist, 3 (1.2%) to a dermatologist, 3 (1.2%) to a gynaecologist, 2 (1.2%) to a nurse practitioner in general practice and 68 patients to a dietitian, mainly because of gastrointestinal complaints. In 116 cases a final diagnosis other than CD was reported. The most commonly reported diagnosis was irritable bowel syndrome (IBS) *n* = 74 (63.8%). In 207 (10.5%) patients of the 1972 individuals who had a gluten related consultation it was reported in the medical records that they followed a (partial) gluten-free diet, in many cases without a reported reason.

### Treatment and follow up

A gluten-free diet was initiated by either the GP or a medical specialist in case of CD. In four cases a follow up plan based on clinical response was noted (i.e. based on a new consultation initiated by the patient because of persisting or recurrent complaints).

## Discussion

In this population-based cohort of 207.200 registered patients, we demonstrated an incidence and prevalence of diagnosed and registered CD in general practice of 0.01% and 0.19%, respectively. In 1296 cases a suspicion of CD led to serological testing. Only 1.6% of these patients tested positive for CD.

Available data on incidence and prevalence on CD in the Netherlands rely on studies that were performed in two large historic population-based cohorts (assembled between 1987–1997) encompassing 50.760 individuals [14]. In this study the prevalence of diagnosed CD was 0.016% (95% CI 0.008–0.031) whereas the prevalence of undiagnosed CD (i.e. undiagnosed patients with positive serology) was 0.35% (95% CI 0.15–0.81) [14]. Although there are data that indicate that the incidence of CD is rising [15], the seroprevalence is very comparable to a more recent study from a closely related geographical region (Germany) [3]. In this cohort of adult individuals (*n* = 8806) the CD prevalence was 0.3 based on tTGA, and endomysium antibodies [3] These data are also in line with observations within the German population by Kratzer and colleagues [16]. Based on serological testing followed up by a duodenal biopsy, they established a CD prevalence of 0.37% [16]. Together, these data indicate that the prevalence of CD in this geographical area of Europe is estimated between 0.3 and 0.4%. and appears substantially lower than the estimated prevalence of 1% frequently referred to in Europe and 1.4% globally [3].

In the current study the prevalence of diagnosed CD was 0.19%. This is a 12-fold higher than Schweizer observed. Partly, this can be explained by the arrival of highly sensitive and specific serological tests and secondly by greater awareness among the general public and physicians since the beginning of the nineties and adaption to a case-finding strategy which has resulted in more CD diagnoses [17].

More importantly, this study conveys the impression that the diagnostic gap may not be as large as implicated in literature and that the diagnostic yield in general practice is better than suspected [3,18].

Thus, where previously published data suggest that for every diagnosed CD case, 5-10 cases remain undiagnosed [14,19] our results and the above mentioned German data suggest that this ratio may liesmore in the order of magnitude of 1 to1.5-2 [14,16]. It should be noted however that we have no recent seroprevalence data available from our population and that such data are warranted to substantiate this thesis. Nevertheless although this is speculative these observations may help to explain the low diagnostic yield of active case finding by GPs through testing for CD as was shown in this study.

This study is the first to explore diagnostic of GPs with respect to CD, based on routine care data. A strength of our study is the use of an extensive database covering more than 200.000 registered patients. Another is our elaborate, CD-sensitive three-track search strategy (Figure 1). Such a search strategy is crucial, as single search strategies all would miss a significant proportion of the target population. Given that almost all individuals in the Netherlands are registered at a GP and they act as gatekeepers it is not probable that a substantial number of patients were missed or double counted. What we have observed is a remarkably high number of gluten related consultations in general practice. Almost 1% of consultations were related to gluten or CD, the majority being from young or middle-aged women. The most common symptoms reported prompting a CD related consultation were gastrointestinal (71.9%). In many of these cases the final diagnosis was IBS. The high frequency of gluten related consultations in combination with our recent observation that the recognition of CD risk factors and knowledge of symptoms/disorders that require serological testing was suboptimal among GPs (unpublished data) suggests that a training program tailored to primary care may increase the diagnostic yield in daily practice.

This study also has limitations that should be mentioned. First, the quality of the data depends on the completeness and accuracy of registration by the GP. However, GPs affiliated to the database that we investigated receive regular training in coding and registration. Relevant contacts may have been missed, which would then have resulted in an underestimation of the actual contact frequency for CD. However, we expect this to be unlikely, owing to our extensive search strategy combining ICPC-1 codes, lab tests and free text mining for CD and gluten relevant search terms. Another limitation of this study is the one year period of the study. A longer historical period might have enabled a more precise estimate of the diagnosed and reported incidence. However, for the main goal of this study, examining the diagnostic behaviour of GPs regarding CD and the diagnostic yield of their diagnostic behaviour, this is inconsequential.

In conclusion we demonstrated a high number of gluten-related consultations in the general practice. Only 1.6% of all patients that were serologically tested for CD actually tested positive. The prevalence of registered diagnosed CD in our cohort of 207.200 registered GP patients was 0.19%. Compared to existing literature, our results suggest that the gap between diagnosed and undiagnosed CD may not be as large as previously stated.
